# Methodological advances in patient-centered rare disease research: the UTHealth Houston Turner Syndrome Society of the United States research registry

**DOI:** 10.1186/s13023-024-03120-1

**Published:** 2024-03-11

**Authors:** Sara Mansoorshahi, Cindy Scurlock, Scientific Advisory Board of the Turner Syndrome Society of the United States Research Registry, Siddharth K Prakash

**Affiliations:** 1 Department of Internal Medicine, John P. and Katherine G. McGovern Medical School, University of Texas Health Science Center at Houston, 6431 Fannin Street, MSB 6.116, Houston, TX 77030 USA; 2 Turner Syndrome Society of the United States, 11250 West Rd, Suite G, Houston, TX 77065 USA

**Keywords:** Turner syndrome, Registry, Patient-centered, Research

## Abstract

**Background:**

Many different clinical specialists provide care to patients with Turner syndrome (TS), who have highly variable clinical manifestations. Therefore, a national TS registry is essential to inform a cohesive approach to healthcare and research. In 2015, the Turner Syndrome Society of the United States (TSSUS) created the Turner Syndrome Research Registry (TSRR) to engage directly with community participants who voluntarily provide longitudinal data about their experiences with TS. TSRR projects are collaborative partnerships between people with TS, TSSUS, and researchers.

**Results:**

To ensure that registry workflows conform to the data privacy choices of participants, TSSUS collaborated with UTHealth Houston in 2021 to create a new version of the TSRR that completely separates participant health data (stored at UTHealth) and personal identifiers (maintained at TSSUS). We developed an innovative Visual Basic (VB) script that, when embedded into Microsoft Outlook, redirects REDCap surveys through TSSUS to participants by matching registry IDs to participant email addresses. Additionally, the utilization of REDCap allows for portability of data as it is an open source platform.

**Conclusion:**

In this report, we will highlight three recent changes that more closely align the TSRR with this mission: a unique and equal collaborative partnership between UTHealth and TSSUS, an open-source platform, REDCap, that ensures data portability and compatibility across institutions, and an innovative survey routing system that retains participant confidentiality without sacrificing REDCap survey distribution capabilities to connect researchers with thousands of participants.

**Supplementary Information:**

The online version contains supplementary material available at 10.1186/s13023-024-03120-1.

## Background

Turner Syndrome (TS) is caused by the absence of all or part of one X chromosome leading to developmental problems such as short stature, ovarian dysfunction, and heart malformations [1, 2]. Because TS involves multiple organ systems, it is often difficult to track the longitudinal trajectories of individuals as they navigate complex healthcare needs [3]. TS is also a rare condition (1 in 2500 females) and requires a coordinated nationwide effort to recruit large cohorts for research studies [4]. Clinical manifestations of TS are highly variable and occur throughout life, requiring long-term natural history studies [5–7]. Many different clinical specialists provide care to TS patients, presenting challenges to a cohesive approach to TS research [8]. Therefore, a national Turner Syndrome registry is essential to inform priorities for healthcare and research.

To address these knowledge gaps, the Turner Syndrome Society of the United States (TSSUS) created the Turner Syndrome Research Registry (TSRR) in 2015 [9]. The purpose of the TSRR is to collect longitudinal data directly from participants about their lifelong experiences with TS. Data from TSRR surveys provides critical feedback about community priorities and facilitates recruitment for research [10].

In 2021, UTHealth Houston partnered with TSSUS to create an improved registry that more closely aligns the TSRR with these priorities. Version 2 of the TSRR is the product of an equal partnership between the TS community, TSSUS, and researchers. The new registry workflows maintain the highest level of data privacy and confidentiality by taking advantage of a lock and key model in which participant data and identifiers are kept separate. The unique REDCap-based structure of the TSRR also facilitates portability of data between participants, investigators, and institutions, fueling the virtuous cycle of TS research. The objectives of this study are to describe the development of new open-source tools that facilitate research communication and data collection while respecting the autonomy and confidentiality of research participants, and to demonstrate the potential of this approach to accelerate rare disease research, as exemplified by the TSRR.

## Methods

The study protocol was reviewed and approved by the Committee for the Protection of Human Subjects at the University of Texas Health Science Center at Houston (IRB# HSC-MS-21-0384). The TSRR is managed using a version of Research Electronic Data Capture (REDCap) that is hosted at the McWilliams School of Biomedical Informatics at UTHealth Houston [11]. REDCap is a secure, web-based application designed to support data capture for research studies, providing (1) an intuitive interface for validated data entry; (2) audit trails for tracking data manipulation and export procedures; (3) automated export procedures for seamless data downloads to common statistical packages; and (4) procedures for importing data from external sources.

The first version of the TSRR was hosted on the Platform for Engaging Everyone Responsibly (PEER, Genetic Alliance, Damascus, MD). To create TSRR version 2, registry data were exported from PEER as a tab-delimited text file, transformed into a data frame using a custom python script, and imported into UTHealth Houston REDCap after quality control and de-identification (Supplemental 1 Data). The initial data included a total of 957 participant records with two instruments: one foundational survey with 73 recoded variables and one instrument with participant contact and privacy preferences.

The REDCap survey routing system was created by installing a custom macro in Microsoft Outlook Visual Basic for Apps (VBA, Supplementary 2 Data). The VBA script redirects any incoming message with a subject line that matches the record ID of a TSRR participant to the corresponding personal email address in a linking log file that is maintained by TSSUS.

## Results

### Enrollment criteria and registry workflows

All eligible participants with a clinical diagnosis of TS or caregivers of someone with TS are invited to enroll in the TSRR through a registration portal on the TSSUS website. Participants complete a foundational survey that requests information about demographics, medical history, and privacy options about their willingness to share their data for different types of research projects. TSSR researchers who wish to access TSRR participants or data must submit research proposals to the TSSUS Scientific Advisory Board (SAB) for review. The role of the SAB is to ensure that the research is of scientific merit and is consistent with the objectives of TSSUS. The SAB then works with researchers to promote selected projects to TSRR participants who consent to be contacted about new studies.

**Fig. 1:**
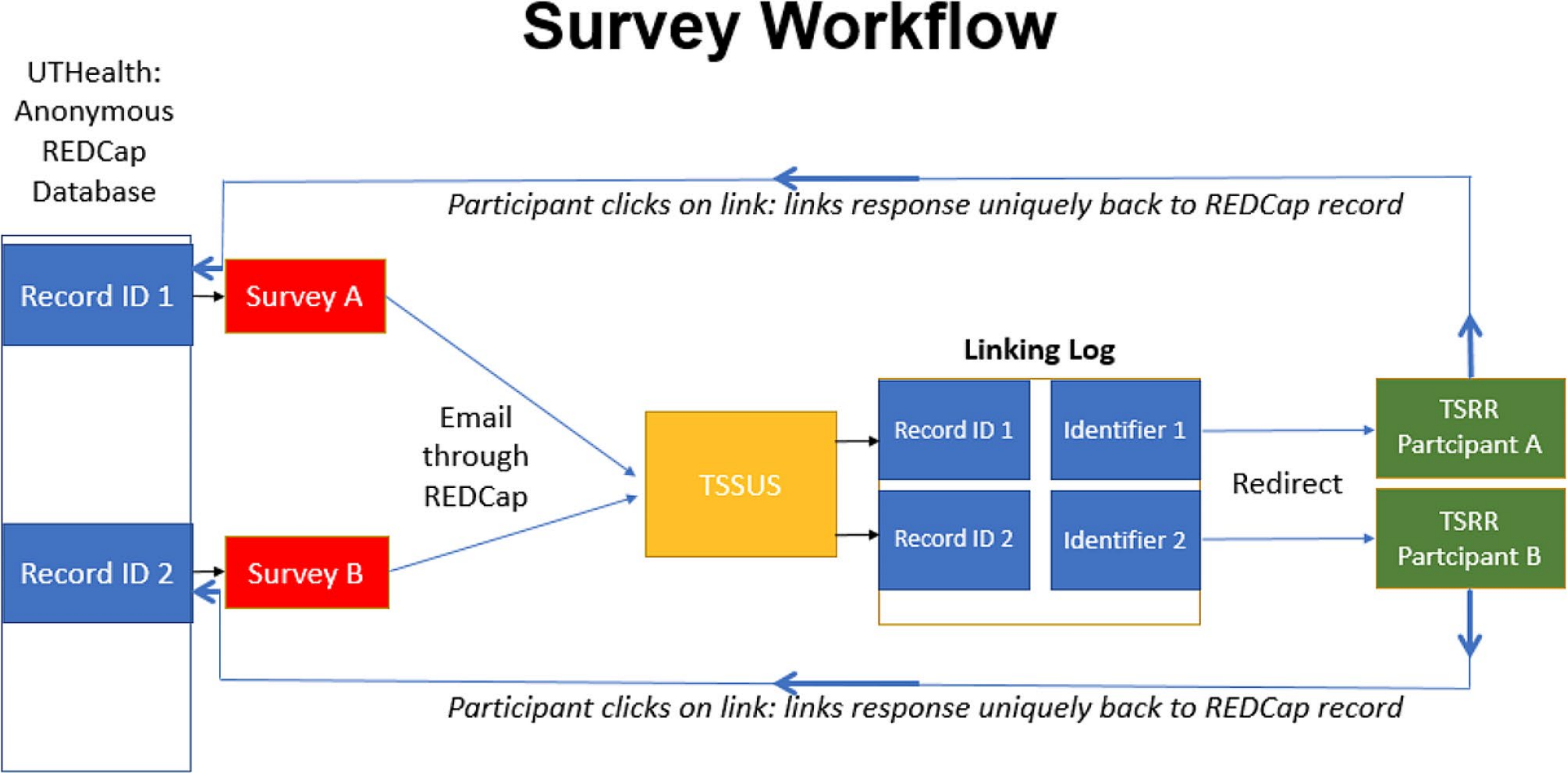
Structure of the Turner Syndrome Research Registry (TSRR). The flow diagram illustrates how surveys can be distributed from the UTHealth Houston REDCap registry to participants, and how self-reported data is returned to REDCap. The Turner Syndrome Society of United States (TSSUS) is the sole gatekeeper of participant information

TSSUS continuously updates a linking log of participant information whenever each new participant registers for the TSRR. Every participant is assigned a global universal identifier (GUID) that consists of pseudonymized data. Each row of the linking log contains the participant identifiers, GUID, and privacy options that were selected by the participant at registration. To complete the registration process, TSSUS exports the GUID and privacy options, but not identifiers, to the UTHealth TSRR team as a templated text file that can be seamlessly imported into REDCap. This workflow ensures that personal identifiers are physically separated from the registry data on two independent encrypted servers. The new REDCap TSRR platform promotes a virtuous cycle that expands the registry with each new project by automating the return of new data back into the registry when new surveys are created (Fig. 1).

### Partnership with TSSUS

The TSRR is the first patient-powered registry that is entirely supported by an equal partnership between a nonprofit organization and an academic institution. TSSUS, the nonprofit organization, hosts all participant identifiers and is the gatekeeper to all interactions with participants, while UTHealth Houston manages the creation and distribution of surveys for TSRR projects. This lock and key model, where both organizations must cooperate in order to maintain the registry, is unique.

The TSRR partnership amplifies the strengths and expertise of each organization and provides flexibility to adapt to changes in data ownership, data use, and privacy requirements. By remaining at the hub of TSRR communications, TSSUS can capitalize on contacts with the TS community that were developed over 30 years, while UTHealth Houston provides research infrastructure and expertise. Researchers who use the customizable REDCap survey system to launch projects benefit from this partnership, while returning new data to the TSRR to fulfill the ‘virtuous cycle.’

### Portability and data security

Data in the TSRR is stored in REDCap instruments. REDCap is an open-source platform that has been widely adopted by academic partners across the United States. Data sharing with collaborators is simple: de-identified data can be exported from one REDCap project and imported into another REDCap project using customized REDCap instruments. REDCap adheres to worldwide data privacy standards such as HIPAA and GDPR [11, 12]. The UTHealth server where TSRR data is stored is encrypted and backed up regularly. Therefore, the REDCap-based structure of the TSRR improves data security and portability to facilitate collaborative research.

The TSRR is easy to sustain if institutions or investigators change because TSRR data can be easily exported to a new site while TSSUS retains complete sovereignty over the data. Data portability is also essential to maintain the virtuous cycle of the TSRR. The virtuous cycle refers to the process whereby researchers return new data or analysis to the TSRR. REDCap data import tools automate this process, removing the need for third-party custodial administration.

### Visual basic survey routing system

We developed an innovative Visual Basic (VB) script that, when embedded into Microsoft Outlook, redirects REDCap surveys through TSSUS to participants by matching registry IDs to participant email addresses (Supplemental Data). Therefore, participant identifiers are never accessible to UTHealth Houston registry support staff or researchers. To facilitate email redirection, we edited the outgoing message settings in the UTHealth TSRR REDCap project so that each participant ID is embedded into the subject line and the recipient email is set to a single address that is monitored by TSSUS personnel. The TSSUS Outlook VB script automatically scans for the participant ID in incoming messages, matches each participant ID to the corresponding email address in the linking log, and redirects outgoing messages to TSRR participants. Because the script automatically links participant IDs to identifiers, thousands of messages can be sent simultaneously to TSRR participants, while TSRR data remains confidential. By editing automated REDCap survey invitation options, emails may be targeted to specific subgroups of recipients that meet prespecified criteria corresponding to variables in the registry such as age, diagnosis, or vital status. The survey distribution tools in REDCap are useful to generate real-time reports about survey completion by TSRR participants.

### Participant enrollment and clinical studies supported by new registry structure

By February 2024, the TSRR had enrolled 1,380 participants and successfully deployed two general health update surveys and three project-specific surveys. The project-specific surveys were designed collaboratively by independent investigators and the TSSUS Scientific Advisory Board to address research priorities that were identified by people with Turner syndrome and stakeholders in the TS community. Eligible TSRR participants who responded to project-specific surveys completed consent forms authorizing study investigators to contact them for interviews or retrieval of health record information. Data from these follow up studies were stored in separate REDCap projects and exported to the TSRR after de-identification. Using this approach, TSRR investigators identified health disparities in access to guideline-recommended medical care for Spanish speakers with TS [13]; characterized the increased prevalence of dermatologic diseases in TS [14]; and described risk factors for postoperative complications after cardiovascular surgeries in young adults with TS, which occur more frequently than expected [15].

## Discussion

### Comparison with insights registry

Inspiring New Science to Guide Healthcare in Turner Syndrome (InsighTS) is a contemporary TS registry that is administered at the University of Colorado Anschutz Medical Campus [16]. InsighTS and the TSRR were intentionally designed to provide complementary snapshots of TS life experiences. InsighTS utilizes a provider-centered model for enrollment and research that primarily relies on data from medical records to build a traditional registry database. In contrast, the TSRR is based on direct survey data from participants that is most appropriate for qualitative research and direct community engagement. Future linkage of these datasets using a shared global universal identifier could eventually provide a more comprehensive longitudinal overview of care navigation and life transitions in TS.

### Limitations

REDCap survey delivery requires TSSUS to maintain an accurate linking log with up-to-date participant contact information. Manual curation of the linking log in the current workflow is time consuming and prone to technical errors. Technical limitations in the current version of the visual basic app prevent sending reminder emails anonymously to survey recipients. We are currently revising the Visual Basic script and TSSUS webform to automate the registration process and optimize survey delivery.

## Conclusion

The TSRR was designed to sustain a virtuous cycle of patient-powered research. In this report, we highlight three recent changes that more closely align the TSRR with this mission: a unique and equal collaborative partnership between UTHealth Houston and TSSUS, an open-source platform, REDCap, that ensures data portability and compatibility across institutions, and an innovative survey routing system that retains participant autonomy and confidentiality without sacrificing REDCap survey distribution capabilities to connect researchers with thousands of participants. TSRR data has catalyzed several projects that were prioritized by TS community stakeholders and jointly developed with the TSSUS Scientific Advisory Board. Cumulative results from these projects will be available to future researchers who apply for TSRR data. This workflow can be configured for a wide variety of robust patient-centered clinical studies, which are particularly valuable for rare disease research.

### Electronic supplementary material

Below is the link to the electronic supplementary material.

Supplementary Material 1

Supplementary Material 2

## Data Availability

Data generated or analyzed during this study are included in this published article and its supplementary information files. If datasets of interest are not included in this study, they are available upon request to the corresponding author, Dr. Siddharth K. Prakash at siddharth.k.prakash@uth.tmc.edu.
