# Supplementary material for: Methodological advances in patient-centered rare disease research: the UTHealth Houston Turner Syndrome Society of the United States research registry
Source: Orphanet J Rare Dis. 2024 Mar 11;19:112. doi: 10.1186/s13023-024-03120-1 (PMC10929126; doi:10.1186/s13023-024-03120-1)
Supplement: Supplementary file 2 — Supplementary Material 2 [file 13023_2024_3120_MOESM2_ESM.docx]

**Steps to deidentify, clean, and transfer PEER data into REDCap to create TSRR version 2:**

1. **Data export from PEER registry: 72,871 lines into TSRR .txt file on 3/11/2021**

PEER data consisted of 951 concatenated surveys with time-stamped multiple choice or yes/no responses.

1. **Extract phenotypes from TSRR .txt file using keywords:**

grep "height" tsrr_files/TSRR.data > height.txt

grep -v "Under" height.txt > height2.txt; mv height2.txt height.txt

grep -v "Over" height.txt > height2.txt; mv height2.txt height.txt

grep -v "System" height.txt > height2.txt; mv height2.txt height.txt

grep "High blood pressure" tsrr_files/TSRR.data > high_blood_pressure.txt

grep "Heart abnormality" tsrr_files/TSRR.data > heart_abnormality.txt

grep "Aortic enlargement" tsrr_files/TSRR.data > aortic_enlargement.txt

grep "Coronary artery disease" tsrr_files/TSRR.data > coronary_artery_disease.txt

grep "High cholesterol" tsrr_files/TSRR.data > high_cholesterol.txt

grep "Kidney abnormality" tsrr_files/TSRR.data > kidney_abnormality.txt

grep "Chronic kidney disease" tsrr_files/TSRR.data > chronic_kidney_disease.txt

grep "Low bone mineral density" tsrr_files/TSRR.data > low_bone_mineral_density.txt

grep "Vitamin D deficiency" tsrr_files/TSRR.data > vitamin_d_deficiency.txt

grep "Curvature of the spine (Scoliosis or Kyphosis)" tsrr_files/TSRR.data > curvature_of_spine.txt

grep "Arthritis" tsrr_files/TSRR.data > arthritis.txt

grep "Osteoporosis" tsrr_files/TSRR.data > osteoporosis.txt

grep "Visual impairment" tsrr_files/TSRR.data > visual_impairment.txt

grep "Hearing impairment" tsrr_files/TSRR.data > hearing_impairment.txt

grep "Chronic ear infections" tsrr_files/TSRR.data > chronic_ear_infections.txt

grep "Ear abnormality" tsrr_files/TSRR.data > ear_abnormality.txt

grep "Craniofacial or dental abnormality" tsrr_files/TSRR.data > craniofacial_or_dental.txt

grep "Ovarian failure (requires estrogen replacement)" tsrr_files/TSRR.data > ovarian_failure.txt

grep "Thyroid disease" tsrr_files/TSRR.data > thyroid_disease.txt

grep "Gastrointestinal (stomach) issues" tsrr_files/TSRR.data > gastrointestinal.txt

grep "Liver disease" tsrr_files/TSRR.data > liver_disease.txt

grep "Diabetes" tsrr_files/TSRR.data > diabetes.txt

grep "Glucose intolerance" tsrr_files/TSRR.data > glucose_intolerance.txt

grep "Short stature or Growth failure" tsrr_files/TSRR.data > short_stature.txt

grep "Lymphedema" tsrr_files/TSRR.data > lymphedema.txt

grep "Anxiety" tsrr_files/TSRR.data > anxiety.txt

grep "Depression" tsrr_files/TSRR.data > depression.txt

grep "Attention Deficit Disorder (with or without hyperactivity)" tsrr_files/TSRR.data > attention_deficit_disorder.txt

grep "Autism Spectrum Disorder" tsrr_files/TSRR.data > autism_spectrum_disorder.txt

grep "Social-Communicative Disorder" tsrr_files/TSRR.data > social_communicative_disorder.txt

grep "Developmental Coordination Disorder" tsrr_files/TSRR.data > developmental_coordination_disorder.txt

grep "Intellectual Disability (Mental retardation)" tsrr_files/TSRR.data > intellectual_disability.txt

grep "Learning Disorder (such as Nonverbal Learning Disorder)" tsrr_files/TSRR.data > learning_disorder.txt

grep "Seizures" tsrr_files/TSRR.data > seizures.txt

grep "Stroke" tsrr_files/TSRR.data > stroke.txt

grep "Pregnancy" tsrr_files/TSRR.data > pregnancy.txt

grep "None of the above" tsrr_files/TSRR.data > none_of_the_above.txt

grep "Cheek swab" tsrr_files/TSRR.data > cheek_swab.txt

grep "Skin chromosome testing" tsrr_files/TSRR.data > skin_chromosome.txt

grep "My doctor" tsrr_files/TSRR.data > informed_doctor.txt

grep "I feel informed" tsrr_files/TSRR.data > informed_me.txt

grep "satisfied" tsrr_files/TSRR.data > satisfied.txt

grep "current weight" tsrr_files/TSRR.data > weight.txt

grep -v "Under" weight.txt > weight2.txt; mv weight2.txt weight.txt

grep -v "Over" weight.txt > weight2.txt; mv weight2.txt weight.txt

grep -v "System" weight.txt > weight2.txt; mv weight2.txt weight.txt

grep "treated with estrogen" tsrr_files/TSRR.data > estrogen_treat.txt

**Steps to deidentify, clean, and transfer PEER data into REDCap data frame (cont):**

grep "years old when I started estrogen" tsrr_files/TSRR.data > estrogen_age.txt

grep "currently receiving estrogen" tsrr_files/TSRR.data > estrogen_current.txt

grep "treated with growth hormone" tsrr_files/TSRR.data > gh_treat.txt

grep "aortic dissection" tsrr_files/TSRR.data > aortic_dissection.txt

grep "self-identified gender" tsrr_files/TSRR.data > gender.txt

grep "chromosomal makeup" tsrr_files/TSRR.data > chromosome_makeup.txt

grep "karyotype" tsrr_files/TSRR.data > karyotype.txt

grep "If you are a parent completing this form" tsrr_files/TSRR.data > parent_for_child.txt

grep "As a parent, I feel informed" tsrr_files/TSRR.data > informed_parent.txt

grep "Bondy" tsrr_files/TSRR.data > bondy_registry.txt

grep "GenTAC" tsrr_files/TSRR.data > gentac_registry.txt

grep "I am interested in taking part" tsrr_files/TSRR.data > interested_research.txt

grep "I am interested in giving" tsrr_files/TSRR.data > give_sample.txt

grep "Bisexual" tsrr_files/TSRR.data > bisexual.txt

grep "Straight" tsrr_files/TSRR.data > straight.txt

grep "Gay or lesbian" tsrr_files/TSRR.data > gay.txt

grep "White" tsrr_files/TSRR.data > white.txt

grep "Asian (i.e." tsrr_files/TSRR.data > asian.txt

grep "Black" tsrr_files/TSRR.data > black.txt

grep "Pacific Islander" tsrr_files/TSRR.data > pacific.txt

grep "Hispanic, Latino" tsrr_files/TSRR.data > latino.txt

grep "American Indian" tsrr_files/TSRR.data > indian.txt

grep "easy to do" tsrr_files/TSRR.data > update_provider.txt

grep "control who saw" tsrr_files/TSRR.data > update_me.txt

grep "Email" tsrr_files/TSRR.data > preferred_email.txt

grep "Phone call" tsrr_files/TSRR.data > preferred_phone.txt

grep "Postal" tsrr_files/TSRR.data > preferred_postal.txt

grep "Text message" tsrr_files/TSRR.data > preferred_text.txt

grep "Aside from this survey" tsrr_files/TSRR.data > other_research.txt

grep "years old when I was diagnosed" tsrr_files/TSRR.data > diagnosis_age.txt

1. **Process phenotype files to create uniform variables:**

awk -F"\t" '{print $4}' height.txt | unexpand -a | awk '{print $1}' > height3.txt

awk -F"\t" '{print $4}' high_blood_pressure.txt | unexpand -a | awk '{print $1}' > high_blood_pressure3.txt

awk -F"\t" '{print $4}' heart_abnormality.txt | unexpand -a | awk '{print $1}' > heart_abnormality3.txt

awk -F"\t" '{print $4}' aortic_enlargement.txt | unexpand -a | awk '{print $1}' > aortic_enlargement3.txt

awk -F"\t" '{print $4}' coronary_artery_disease.txt | unexpand -a | awk '{print $1}' > coronary_artery_disease3.txt

awk -F"\t" '{print $4}' high_cholesterol.txt | unexpand -a | awk '{print $1}' > high_cholesterol3.txt

awk -F"\t" '{print $4}' kidney_abnormality.txt | unexpand -a | awk '{print $1}' > kidney_abnormality3.txt

awk -F"\t" '{print $4}' chronic_kidney_disease.txt | unexpand -a | awk '{print $1}' > chronic_kidney_disease3.txt

awk -F"\t" '{print $4}' low_bone_mineral_density.txt | unexpand -a | awk '{print $1}' > low_bone_mineral_density3.txt

awk -F"\t" '{print $4}' vitamin_d_deficiency.txt | unexpand -a | awk '{print $1}' > vitamin_d_deficiency3.txt

awk -F"\t" '{print $4}' curvature_of_spine.txt | unexpand -a | awk '{print $1}' > curvature_of_spine3.txt

awk -F"\t" '{print $4}' arthritis.txt | unexpand -a | awk '{print $1}' > arthritis3.txt

awk -F"\t" '{print $4}' osteoporosis.txt | unexpand -a | awk '{print $1}' > osteoporosis3.txt

awk -F"\t" '{print $4}' visual_impairment.txt | unexpand -a | awk '{print $1}' > visual_impairment3.txt

awk -F"\t" '{print $4}' hearing_impairment.txt | unexpand -a | awk '{print $1}' > hearing_impairment3.txt

awk -F"\t" '{print $4}' chronic_ear_infections.txt | unexpand -a | awk '{print $1}' > chronic_ear_infections3.txt

awk -F"\t" '{print $4}' ear_abnormality.txt | unexpand -a | awk '{print $1}' > ear_abnormality3.txt

awk -F"\t" '{print $4}' craniofacial_or_dental.txt | unexpand -a | awk '{print $1}' > craniofacial_or_dental3.txt

awk -F"\t" '{print $4}' ovarian_failure.txt | unexpand -a | awk '{print $1}' > ovarian_failure3.txt

awk -F"\t" '{print $4}' thyroid_disease.txt | unexpand -a | awk '{print $1}' > thyroid_disease3.txt

**Steps to deidentify, clean, and transfer PEER data into REDCap data frame (cont):**

awk -F"\t" '{print $4}' gastrointestinal.txt | unexpand -a | awk '{print $1}' > gastrointestinal3.txt

awk -F"\t" '{print $4}' liver_disease.txt | unexpand -a | awk '{print $1}' > liver_disease3.txt

awk -F"\t" '{print $4}' diabetes.txt | unexpand -a | awk '{print $1}' > diabetes3.txt

awk -F"\t" '{print $4}' glucose_intolerance.txt | unexpand -a | awk '{print $1}' > glucose_intolerance3.txt

awk -F"\t" '{print $4}' short_stature.txt | unexpand -a | awk '{print $1}' > short_stature3.txt

awk -F"\t" '{print $4}' lymphedema.txt | unexpand -a | awk '{print $1}' > lymphedema3.txt

awk -F"\t" '{print $4}' anxiety.txt | unexpand -a | awk '{print $1}' > anxiety3.txt

awk -F"\t" '{print $4}' depression.txt | unexpand -a | awk '{print $1}' > depression3.txt

awk -F"\t" '{print $4}' attention_deficit_disorder.txt | unexpand -a | awk '{print $1}' > attention_deficit_disorder3.txt

awk -F"\t" '{print $4}' autism_spectrum_disorder.txt | unexpand -a | awk '{print $1}' > autism_spectrum_disorder3.txt

awk -F"\t" '{print $4}' social_communicative_disorder.txt | unexpand -a | awk '{print $1}' > social_communicative_disorder3.txt

awk -F"\t" '{print $4}' developmental_coordination_disorder.txt | unexpand -a | awk '{print $1}' > developmental_coordination_disorder3.txt

awk -F"\t" '{print $4}' intellectual_disability.txt | unexpand -a | awk '{print $1}' > intellectual_disability3.txt

awk -F"\t" '{print $4}' learning_disorder.txt | unexpand -a | awk '{print $1}' > learning_disorder3.txt

awk -F"\t" '{print $4}' seizures.txt | unexpand -a | awk '{print $1}' > seizures3.txt

awk -F"\t" '{print $4}' stroke.txt | unexpand -a | awk '{print $1}' > stroke3.txt

awk -F"\t" '{print $4}' pregnancy.txt | unexpand -a | awk '{print $1}' > pregnancy3.txt

awk -F"\t" '{print $4}' none_of_the_above.txt | unexpand -a | awk '{print $1}' > none_of_the_above3.txt

awk -F"\t" '{print $4}' cheek_swab.txt | unexpand -a | awk '{print $1}' > cheek_swab3.txt

awk -F"\t" '{print $4}' diagnosis_age.txt | unexpand -a | awk '{print $1}' > diagnosis_age3.txt

awk -F"\t" '{print $4}' update_me.txt | unexpand -a | awk '{print $1}' > update_me3.txt

awk -F"\t" '{print $4}' informed_doctor.txt | unexpand -a | awk '{print $1}' > informed_doctor3.txt

awk -F"\t" '{print $4}' satisfied.txt | unexpand -a | awk '{print $1}' > satisfied3.txt

awk -F"\t" '{print $4}' estrogen_treat.txt | unexpand -a | awk '{print $1}' > estrogen_treat3.txt

awk -F"\t" '{print $4}' estrogen_age.txt | unexpand -a | awk '{print $1}' > estrogen_age3.txt

awk -F"\t" '{print $4}' estrogen_current.txt | unexpand -a | awk '{print $1}' > estrogen_current3.txt

awk -F"\t" '{print $4}' gh_treat.txt | unexpand -a | awk '{print $1}' > gh_treat3.txt

awk -F"\t" '{print $4}' aortic_dissection.txt | unexpand -a | awk '{print $1}' > aortic_dissection3.txt

awk -F"\t" '{print $4}' gender.txt | unexpand -a | awk '{print $1}' > gender3.txt

awk -F"\t" '{print $4}' chromosome_makeup.txt | unexpand -a | awk '{print $1}' > chromosome_makeup3.txt

awk -F"\t" '{print $4}' other_research.txt | unexpand -a | awk '{print $1}' > other_research3.txt

awk -F"\t" '{print $4}' karyotype.txt | unexpand -a | awk '{print $1}' > karyotype3.txt

awk -F"\t" '{print $4}' parent_for_child.txt | unexpand -a | awk '{print $1}' > parent_for_child3.txt

awk -F"\t" '{print $4}' informed_parent.txt | unexpand -a | awk '{print $1}' > informed_parent3.txt

awk -F"\t" '{print $4}' weight.txt | unexpand -a | awk '{print $1}' > weight3.txt

cut -f1,8 height.txt > height2.txt

cut -f1,8 high_blood_pressure.txt > high_blood_pressure2.txt

cut -f1,8 heart_abnormality.txt > heart_abnormality2.txt

cut -f1,8 aortic_enlargement.txt > aortic_enlargement2.txt

cut -f1,8 coronary_artery_disease.txt > coronary_artery_disease2.txt

cut -f1,8 high_cholesterol.txt > high_cholesterol2.txt

cut -f1,8 kidney_abnormality.txt > kidney_abnormality2.txt

cut -f1,8 chronic_kidney_disease.txt > chronic_kidney_disease2.txt

cut -f1,8 low_bone_mineral_density.txt > low_bone_mineral_density2.txt

cut -f1,8 vitamin_d_deficiency.txt > vitamin_d_deficiency2.txt

cut -f1,8 curvature_of_spine.txt > curvature_of_spine2.txt

cut -f1,8 arthritis.txt > arthritis2.txt

cut -f1,8 osteoporosis.txt > osteoporosis2.txt

cut -f1,8 visual_impairment.txt > visual_impairment2.txt

**Steps to deidentify, clean, and transfer PEER data into REDCap data frame (cont):**

cut -f1,8 hearing_impairment.txt > hearing_impairment2.txt

cut -f1,8 chronic_ear_infections.txt > chronic_ear_infections2.txt

cut -f1,8 ear_abnormality.txt > ear_abnormality2.txt

cut -f1,8 craniofacial_or_dental.txt > craniofacial_or_dental2.txt

cut -f1,8 ovarian_failure.txt > ovarian_failure2.txt

cut -f1,8 thyroid_disease.txt > thyroid_disease2.txt

cut -f1,8 gastrointestinal.txt > gastrointestinal2.txt

cut -f1,8 liver_disease.txt > liver_disease2.txt

cut -f1,8 diabetes.txt > diabetes2.txt

cut -f1,8 glucose_intolerance.txt > glucose_intolerance2.txt

cut -f1,8 short_stature.txt > short_stature2.txt

cut -f1,8 lymphedema.txt > lymphedema2.txt

cut -f1,8 anxiety.txt > anxiety2.txt

cut -f1,8 depression.txt > depression2.txt

cut -f1,8 attention_deficit_disorder.txt > attention_deficit_disorder2.txt

cut -f1,8 autism_spectrum_disorder.txt > autism_spectrum_disorder2.txt

cut -f1,8 social_communicative_disorder.txt > social_communicative_disorder2.txt

cut -f1,8 developmental_coordination_disorder.txt > developmental_coordination_disorder2.txt

cut -f1,8 intellectual_disability.txt > intellectual_disability2.txt

cut -f1,8 learning_disorder.txt > learning_disorder2.txt

cut -f1,8 seizures.txt > seizures2.txt

cut -f1,8 stroke.txt > stroke2.txt

cut -f1,8 pregnancy.txt > pregnancy2.txt

cut -f1,8 none_of_the_above.txt > none_of_the_above2.txt

cut -f1,8 cheek_swab.txt > cheek_swab2.txt

cut -f1,8 diagnosis_age.txt > diagnosis_age2.txt

cut -f1,8 update_me.txt > update_me2.txt

cut -f1,8 informed_doctor.txt > informed_doctor2.txt

cut -f1,8 satisfied.txt > satisfied2.txt

cut -f1,8 estrogen_treat.txt > estrogen_treat2.txt

cut -f1,8 estrogen_age.txt > estrogen_age2.txt

cut -f1,8 estrogen_current.txt > estrogen_current2.txt

cut -f1,8 gh_treat.txt > gh_treat2.txt

cut -f1,8 aortic_dissection.txt > aortic_dissection2.txt

cut -f1,8 gender.txt > gender2.txt

cut -f1,8 chromosome_makeup.txt > chromosome_makeup2.txt

cut -f1,8 other_research.txt > other_research2.txt

cut -f1,8 karyotype.txt > karyotype2.txt

cut -f1,8 parent_for_child.txt > parent_for_child2.txt

cut -f1,8 informed_parent.txt > informed_parent2.txt

cut -f1,8 weight.txt > weight2.txt

paste height3.txt height2.txt | awk -F"\t" 'BEGIN {OFS="\t"}{print $2,$1,$3}' > height4.txt

paste high_blood_pressure3.txt high_blood_pressure2.txt | awk -F"\t" 'BEGIN {OFS="\t"}{print $2,$1,$3}' > high_blood_pressure4.txt

paste heart_abnormality3.txt heart_abnormality2.txt | awk -F"\t" 'BEGIN {OFS="\t"}{print $2,$1,$3}' > heart_abnormality4.txt

paste aortic_enlargement3.txt aortic_enlargement2.txt | awk -F"\t" 'BEGIN {OFS="\t"}{print $2,$1,$3}' > aortic_enlargement4.txt

paste coronary_artery_disease3.txt coronary_artery_disease2.txt | awk -F"\t" 'BEGIN {OFS="\t"}{print $2,$1,$3}' > coronary_artery_disease4.txt

paste high_cholesterol3.txt high_cholesterol2.txt | awk -F"\t" 'BEGIN {OFS="\t"}{print $2,$1,$3}' > high_cholesterol4.txt

**Steps to deidentify, clean, and transfer PEER data into REDCap data frame (cont):**

paste kidney_abnormality3.txt kidney_abnormality2.txt | awk -F"\t" 'BEGIN {OFS="\t"}{print $2,$1,$3}' > kidney_abnormality4.txt

paste chronic_kidney_disease3.txt chronic_kidney_disease2.txt | awk -F"\t" 'BEGIN {OFS="\t"}{print $2,$1,$3}' > chronic_kidney_disease4.txt

paste low_bone_mineral_density3.txt low_bone_mineral_density2.txt | awk -F"\t" 'BEGIN {OFS="\t"}{print $2,$1,$3}' > low_bone_mineral_density4.txt

paste vitamin_d_deficiency3.txt vitamin_d_deficiency2.txt | awk -F"\t" 'BEGIN {OFS="\t"}{print $2,$1,$3}' > vitamin_d_deficiency4.txt

paste curvature_of_spine3.txt curvature_of_spine2.txt | awk -F"\t" 'BEGIN {OFS="\t"}{print $2,$1,$3}' > curvature_of_spine4.txt

paste arthritis3.txt arthritis2.txt | awk -F"\t" 'BEGIN {OFS="\t"}{print $2,$1,$3}' > arthritis4.txt

paste osteoporosis3.txt osteoporosis2.txt | awk -F"\t" 'BEGIN {OFS="\t"}{print $2,$1,$3}' > osteoporosis4.txt

paste visual_impairment3.txt visual_impairment2.txt | awk -F"\t" 'BEGIN {OFS="\t"}{print $2,$1,$3}' > visual_impairment4.txt

paste hearing_impairment3.txt hearing_impairment2.txt | awk -F"\t" 'BEGIN {OFS="\t"}{print $2,$1,$3}' > hearing_impairment4.txt

paste chronic_ear_infections3.txt chronic_ear_infections2.txt | awk -F"\t" 'BEGIN {OFS="\t"}{print $2,$1,$3}' > chronic_ear_infections4.txt

paste ear_abnormality3.txt ear_abnormality2.txt | awk -F"\t" 'BEGIN {OFS="\t"}{print $2,$1,$3}' > ear_abnormality4.txt

paste craniofacial_or_dental3.txt craniofacial_or_dental2.txt | awk -F"\t" 'BEGIN {OFS="\t"}{print $2,$1,$3}' > craniofacial_or_dental4.txt

paste ovarian_failure3.txt ovarian_failure2.txt | awk -F"\t" 'BEGIN {OFS="\t"}{print $2,$1,$3}' > ovarian_failure4.txt

paste thyroid_disease3.txt thyroid_disease2.txt | awk -F"\t" 'BEGIN {OFS="\t"}{print $2,$1,$3}' > thyroid_disease4.txt

paste gastrointestinal3.txt gastrointestinal2.txt | awk -F"\t" 'BEGIN {OFS="\t"}{print $2,$1,$3}' > gastrointestinal4.txt

paste liver_disease3.txt liver_disease2.txt | awk -F"\t" 'BEGIN {OFS="\t"}{print $2,$1,$3}' > liver_disease4.txt

paste diabetes3.txt diabetes2.txt | awk -F"\t" 'BEGIN {OFS="\t"}{print $2,$1,$3}' > diabetes4.txt

paste glucose_intolerance3.txt glucose_intolerance2.txt | awk -F"\t" 'BEGIN {OFS="\t"}{print $2,$1,$3}' > glucose_intolerance4.txt

paste short_stature3.txt short_stature2.txt | awk -F"\t" 'BEGIN {OFS="\t"}{print $2,$1,$3}' > short_stature4.txt

paste lymphedema3.txt lymphedema2.txt | awk -F"\t" 'BEGIN {OFS="\t"}{print $2,$1,$3}' > lymphedema4.txt

paste anxiety3.txt anxiety2.txt | awk -F"\t" 'BEGIN {OFS="\t"}{print $2,$1,$3}' > anxiety4.txt

paste depression3.txt depression2.txt | awk -F"\t" 'BEGIN {OFS="\t"}{print $2,$1,$3}' > depression4.txt

paste attention_deficit_disorder3.txt attention_deficit_disorder2.txt | awk -F"\t" 'BEGIN {OFS="\t"}{print $2,$1,$3}' > attention_deficit_disorder4.txt

paste autism_spectrum_disorder3.txt autism_spectrum_disorder2.txt | awk -F"\t" 'BEGIN {OFS="\t"}{print $2,$1,$3}' > autism_spectrum_disorder4.txt

paste social_communicative_disorder3.txt social_communicative_disorder2.txt | awk -F"\t" 'BEGIN {OFS="\t"}{print $2,$1,$3}' > social_communicative_disorder4.txt

paste developmental_coordination_disorder3.txt developmental_coordination_disorder2.txt | awk -F"\t" 'BEGIN {OFS="\t"}{print $2,$1,$3}' > developmental_coordination_disorder4.txt

paste intellectual_disability3.txt intellectual_disability2.txt | awk -F"\t" 'BEGIN {OFS="\t"}{print $2,$1,$3}' > intellectual_disability4.txt

paste learning_disorder3.txt learning_disorder2.txt | awk -F"\t" 'BEGIN {OFS="\t"}{print $2,$1,$3}' > learning_disorder4.txt

paste seizures3.txt seizures2.txt | awk -F"\t" 'BEGIN {OFS="\t"}{print $2,$1,$3}' > seizures4.txt

paste stroke3.txt stroke2.txt | awk -F"\t" 'BEGIN {OFS="\t"}{print $2,$1,$3}' > stroke4.txt

paste pregnancy3.txt pregnancy2.txt | awk -F"\t" 'BEGIN {OFS="\t"}{print $2,$1,$3}' > pregnancy4.txt

paste none_of_the_above3.txt none_of_the_above2.txt | awk -F"\t" 'BEGIN {OFS="\t"}{print $2,$1,$3}' > none_of_the_above4.txt

paste cheek_swab3.txt cheek_swab2.txt | awk -F"\t" 'BEGIN {OFS="\t"}{print $2,$1,$3}' > cheek_swab4.txt

paste diagnosis_age3.txt diagnosis_age2.txt | awk -F"\t" 'BEGIN {OFS="\t"}{print $2,$1,$3}' > diagnosis_age4.txt

paste update_me3.txt update_me2.txt | awk -F"\t" 'BEGIN {OFS="\t"}{print $2,$1,$3}' > update_me4.txt

**Steps to deidentify, clean, and transfer PEER data into REDCap data frame (cont):**

paste informed_doctor3.txt informed_doctor2.txt | awk -F"\t" 'BEGIN {OFS="\t"}{print $2,$1,$3}' > informed_doctor4.txt

paste satisfied3.txt satisfied2.txt | awk -F"\t" 'BEGIN {OFS="\t"}{print $2,$1,$3}' > satisfied4.txt

paste estrogen_treat3.txt estrogen_treat2.txt | awk -F"\t" 'BEGIN {OFS="\t"}{print $2,$1,$3}' > estrogen_treat4.txt

paste estrogen_age3.txt estrogen_age2.txt | awk -F"\t" 'BEGIN {OFS="\t"}{print $2,$1,$3}' > estrogen_age4.txt

paste estrogen_current3.txt estrogen_current2.txt | awk -F"\t" 'BEGIN {OFS="\t"}{print $2,$1,$3}' > estrogen_current4.txt

paste gh_treat3.txt gh_treat2.txt | awk -F"\t" 'BEGIN {OFS="\t"}{print $2,$1,$3}' > gh_treat4.txt

paste aortic_dissection3.txt aortic_dissection2.txt | awk -F"\t" 'BEGIN {OFS="\t"}{print $2,$1,$3}' > aortic_dissection4.txt

paste gender3.txt gender2.txt | awk -F"\t" 'BEGIN {OFS="\t"}{print $2,$1,$3}' > gender4.txt

paste chromosome_makeup3.txt chromosome_makeup2.txt | awk -F"\t" 'BEGIN {OFS="\t"}{print $2,$1,$3}' > chromosome_makeup4.txt

paste other_research3.txt other_research2.txt | awk -F"\t" 'BEGIN {OFS="\t"}{print $2,$1,$3}' > other_research4.txt

paste karyotype3.txt karyotype2.txt | awk -F"\t" 'BEGIN {OFS="\t"}{print $2,$1,$3}' > karyotype4.txt

paste parent_for_child3.txt parent_for_child2.txt | awk -F"\t" 'BEGIN {OFS="\t"}{print $2,$1,$3}' > parent_for_child4.txt

paste informed_parent3.txt informed_parent2.txt | awk -F"\t" 'BEGIN {OFS="\t"}{print $2,$1,$3}' > informed_parent4.txt

paste weight3.txt weight2.txt | awk -F"\t" 'BEGIN {OFS="\t"}{print $2,$1,$3}' > weight4.txt

awk -F"\t" 'BEGIN {OFS="\t"} {$1=$1"#"$2;print $1,$3}' height4.txt > height2.txt ;mv height2.txt height.txt

awk -F"\t" 'BEGIN {OFS="\t"} {$1=$1"#"$2;print $1,$3}' high_blood_pressure4.txt > high_blood_pressure2.txt ;mv high_blood_pressure2.txt high_blood_pressure.txt

awk -F"\t" 'BEGIN {OFS="\t"} {$1=$1"#"$2;print $1,$3}' heart_abnormality4.txt > heart_abnormality2.txt ;mv heart_abnormality2.txt heart_abnormality.txt

awk -F"\t" 'BEGIN {OFS="\t"} {$1=$1"#"$2;print $1,$3}' aortic_enlargement4.txt > aortic_enlargement2.txt ;mv aortic_enlargement2.txt aortic_enlargement.txt

awk -F"\t" 'BEGIN {OFS="\t"} {$1=$1"#"$2;print $1,$3}' coronary_artery_disease4.txt > coronary_artery_disease2.txt ;mv coronary_artery_disease2.txt coronary_artery_disease.txt

awk -F"\t" 'BEGIN {OFS="\t"} {$1=$1"#"$2;print $1,$3}' high_cholesterol4.txt > high_cholesterol2.txt ;mv high_cholesterol2.txt high_cholesterol.txt

awk -F"\t" 'BEGIN {OFS="\t"} {$1=$1"#"$2;print $1,$3}' kidney_abnormality4.txt > kidney_abnormality2.txt ;mv kidney_abnormality2.txt kidney_abnormality.txt

awk -F"\t" 'BEGIN {OFS="\t"} {$1=$1"#"$2;print $1,$3}' chronic_kidney_disease4.txt > chronic_kidney_disease2.txt ;mv chronic_kidney_disease2.txt chronic_kidney_disease.txt

awk -F"\t" 'BEGIN {OFS="\t"} {$1=$1"#"$2;print $1,$3}' low_bone_mineral_density4.txt > low_bone_mineral_density2.txt ;mv low_bone_mineral_density2.txt low_bone_mineral_density.txt

awk -F"\t" 'BEGIN {OFS="\t"} {$1=$1"#"$2;print $1,$3}' vitamin_d_deficiency4.txt > vitamin_d_deficiency2.txt ;mv vitamin_d_deficiency2.txt vitamin_d_deficiency.txt

awk -F"\t" 'BEGIN {OFS="\t"} {$1=$1"#"$2;print $1,$3}' curvature_of_spine4.txt > curvature_of_spine2.txt ;mv curvature_of_spine2.txt curvature_of_spine.txt

awk -F"\t" 'BEGIN {OFS="\t"} {$1=$1"#"$2;print $1,$3}' arthritis4.txt > arthritis2.txt ;mv arthritis2.txt arthritis.txt

awk -F"\t" 'BEGIN {OFS="\t"} {$1=$1"#"$2;print $1,$3}' osteoporosis4.txt > osteoporosis2.txt ;mv osteoporosis2.txt osteoporosis.txt

awk -F"\t" 'BEGIN {OFS="\t"} {$1=$1"#"$2;print $1,$3}' visual_impairment4.txt > visual_impairment2.txt ;mv visual_impairment2.txt visual_impairment.txt

awk -F"\t" 'BEGIN {OFS="\t"} {$1=$1"#"$2;print $1,$3}' hearing_impairment4.txt > hearing_impairment2.txt ;mv hearing_impairment2.txt hearing_impairment.txt

awk -F"\t" 'BEGIN {OFS="\t"} {$1=$1"#"$2;print $1,$3}' chronic_ear_infections4.txt > chronic_ear_infections2.txt ;mv chronic_ear_infections2.txt chronic_ear_infections.txt

awk -F"\t" 'BEGIN {OFS="\t"} {$1=$1"#"$2;print $1,$3}' ear_abnormality4.txt > ear_abnormality2.txt ;mv ear_abnormality2.txt ear_abnormality.txt

awk -F"\t" 'BEGIN {OFS="\t"} {$1=$1"#"$2;print $1,$3}' craniofacial_or_dental4.txt > craniofacial_or_dental2.txt ;mv craniofacial_or_dental2.txt craniofacial_or_dental.txt

awk -F"\t" 'BEGIN {OFS="\t"} {$1=$1"#"$2;print $1,$3}' ovarian_failure4.txt > ovarian_failure2.txt ;mv ovarian_failure2.txt ovarian_failure.txt

**Steps to deidentify, clean, and transfer PEER data into REDCap data frame (cont):**

awk -F"\t" 'BEGIN {OFS="\t"} {$1=$1"#"$2;print $1,$3}' thyroid_disease4.txt > thyroid_disease2.txt ;mv thyroid_disease2.txt thyroid_disease.txt

awk -F"\t" 'BEGIN {OFS="\t"} {$1=$1"#"$2;print $1,$3}' gastrointestinal4.txt > gastrointestinal2.txt ;mv gastrointestinal2.txt gastrointestinal.txt

awk -F"\t" 'BEGIN {OFS="\t"} {$1=$1"#"$2;print $1,$3}' liver_disease4.txt > liver_disease2.txt ;mv liver_disease2.txt liver_disease.txt

awk -F"\t" 'BEGIN {OFS="\t"} {$1=$1"#"$2;print $1,$3}' diabetes4.txt > diabetes2.txt ;mv diabetes2.txt diabetes.txt

awk -F"\t" 'BEGIN {OFS="\t"} {$1=$1"#"$2;print $1,$3}' glucose_intolerance4.txt > glucose_intolerance2.txt ;mv glucose_intolerance2.txt glucose_intolerance.txt

awk -F"\t" 'BEGIN {OFS="\t"} {$1=$1"#"$2;print $1,$3}' short_stature4.txt > short_stature2.txt ;mv short_stature2.txt short_stature.txt

awk -F"\t" 'BEGIN {OFS="\t"} {$1=$1"#"$2;print $1,$3}' lymphedema4.txt > lymphedema2.txt ;mv lymphedema2.txt lymphedema.txt

awk -F"\t" 'BEGIN {OFS="\t"} {$1=$1"#"$2;print $1,$3}' anxiety4.txt > anxiety2.txt ;mv anxiety2.txt anxiety.txt

awk -F"\t" 'BEGIN {OFS="\t"} {$1=$1"#"$2;print $1,$3}' depression4.txt > depression2.txt ;mv depression2.txt depression.txt

awk -F"\t" 'BEGIN {OFS="\t"} {$1=$1"#"$2;print $1,$3}' attention_deficit_disorder4.txt > attention_deficit_disorder2.txt ;mv attention_deficit_disorder2.txt attention_deficit_disorder.txt

awk -F"\t" 'BEGIN {OFS="\t"} {$1=$1"#"$2;print $1,$3}' autism_spectrum_disorder4.txt > autism_spectrum_disorder2.txt ;mv autism_spectrum_disorder2.txt autism_spectrum_disorder.txt

awk -F"\t" 'BEGIN {OFS="\t"} {$1=$1"#"$2;print $1,$3}' social_communicative_disorder4.txt > social_communicative_disorder2.txt ;mv social_communicative_disorder2.txt social_communicative_disorder.txt

awk -F"\t" 'BEGIN {OFS="\t"} {$1=$1"#"$2;print $1,$3}' developmental_coordination_disorder4.txt > developmental_coordination_disorder2.txt ;mv developmental_coordination_disorder2.txt developmental_coordination_disorder.txt

awk -F"\t" 'BEGIN {OFS="\t"} {$1=$1"#"$2;print $1,$3}' intellectual_disability4.txt > intellectual_disability2.txt ;mv intellectual_disability2.txt intellectual_disability.txt

awk -F"\t" 'BEGIN {OFS="\t"} {$1=$1"#"$2;print $1,$3}' learning_disorder4.txt > learning_disorder2.txt ;mv learning_disorder2.txt learning_disorder.txt

awk -F"\t" 'BEGIN {OFS="\t"} {$1=$1"#"$2;print $1,$3}' seizures4.txt > seizures2.txt ;mv seizures2.txt seizures.txt

awk -F"\t" 'BEGIN {OFS="\t"} {$1=$1"#"$2;print $1,$3}' stroke4.txt > stroke2.txt ;mv stroke2.txt stroke.txt

awk -F"\t" 'BEGIN {OFS="\t"} {$1=$1"#"$2;print $1,$3}' pregnancy4.txt > pregnancy2.txt ;mv pregnancy2.txt pregnancy.txt

awk -F"\t" 'BEGIN {OFS="\t"} {$1=$1"#"$2;print $1,$3}' none_of_the_above4.txt > none_of_the_above2.txt ;mv none_of_the_above2.txt none_of_the_above.txt

awk -F"\t" 'BEGIN {OFS="\t"} {$1=$1"#"$2;print $1,$3}' cheek_swab4.txt > cheek_swab2.txt ;mv cheek_swab2.txt cheek_swab.txt

awk -F"\t" 'BEGIN {OFS="\t"} {$1=$1"#"$2;print $1,$3}' diagnosis_age4.txt > diagnosis_age2.txt ;mv diagnosis_age2.txt diagnosis_age.txt

awk -F"\t" 'BEGIN {OFS="\t"} {$1=$1"#"$2;print $1,$3}' update_me4.txt > update_me2.txt ;mv update_me2.txt update_me.txt

awk -F"\t" 'BEGIN {OFS="\t"} {$1=$1"#"$2;print $1,$3}' informed_doctor4.txt > informed_doctor2.txt ;mv informed_doctor2.txt informed_doctor.txt

awk -F"\t" 'BEGIN {OFS="\t"} {$1=$1"#"$2;print $1,$3}' satisfied4.txt > satisfied2.txt ;mv satisfied2.txt satisfied.txt

awk -F"\t" 'BEGIN {OFS="\t"} {$1=$1"#"$2;print $1,$3}' estrogen_treat4.txt > estrogen_treat2.txt ;mv estrogen_treat2.txt estrogen_treat.txt

awk -F"\t" 'BEGIN {OFS="\t"} {$1=$1"#"$2;print $1,$3}' estrogen_age4.txt > estrogen_age2.txt ;mv estrogen_age2.txt estrogen_age.txt

awk -F"\t" 'BEGIN {OFS="\t"} {$1=$1"#"$2;print $1,$3}' estrogen_current4.txt > estrogen_current2.txt ;mv estrogen_current2.txt estrogen_current.txt

awk -F"\t" 'BEGIN {OFS="\t"} {$1=$1"#"$2;print $1,$3}' gh_treat4.txt > gh_treat2.txt ;mv gh_treat2.txt gh_treat.txt

**Steps to deidentify, clean, and transfer PEER data into REDCap (cont):**

awk -F"\t" 'BEGIN {OFS="\t"} {$1=$1"#"$2;print $1,$3}' aortic_dissection4.txt > aortic_dissection2.txt ;mv aortic_dissection2.txt aortic_dissection.txt

awk -F"\t" 'BEGIN {OFS="\t"} {$1=$1"#"$2;print $1,$3}' gender4.txt > gender2.txt ;mv gender2.txt gender.txt

awk -F"\t" 'BEGIN {OFS="\t"} {$1=$1"#"$2;print $1,$3}' chromosome_makeup4.txt > chromosome_makeup2.txt ;mv chromosome_makeup2.txt chromosome_makeup.txt

awk -F"\t" 'BEGIN {OFS="\t"} {$1=$1"#"$2;print $1,$3}' other_research4.txt > other_research2.txt ;mv other_research2.txt other_research.txt

awk -F"\t" 'BEGIN {OFS="\t"} {$1=$1"#"$2;print $1,$3}' karyotype4.txt > karyotype2.txt ;mv karyotype2.txt karyotype.txt

awk -F"\t" 'BEGIN {OFS="\t"} {$1=$1"#"$2;print $1,$3}' parent_for_child4.txt > parent_for_child2.txt ;mv parent_for_child2.txt parent_for_child.txt

awk -F"\t" 'BEGIN {OFS="\t"} {$1=$1"#"$2;print $1,$3}' informed_parent4.txt > informed_parent2.txt ;mv informed_parent2.txt informed_parent.txt

awk -F"\t" 'BEGIN {OFS="\t"} {$1=$1"#"$2;print $1,$3}' weight4.txt > weight2.txt ;mv weight2.txt weight.txt

awk -F"\t" '{print $4}' bondy_registry.txt | unexpand -a | awk '{print $1}' > bondy_registry3.txt

cut -f1,8 bondy_registry.txt > bondy_registry2.txt

paste bondy_registry3.txt bondy_registry2.txt | awk -F"\t" 'BEGIN {OFS="\t"}{print $2,$1,$3}' > bondy_registry4.txt

awk -F"\t" 'BEGIN {OFS="\t"} {$1=$1"#"$2;print $1,$3}' bondy_registry4.txt > bondy_registry2.txt ;mv bondy_registry2.txt bondy_registry.txt

awk -F"\t" '{print $4}' white.txt | unexpand -a | awk '{print $1}' > white3.txt

cut -f1,8 white.txt > white2.txt

paste white3.txt white2.txt | awk -F"\t" 'BEGIN {OFS="\t"}{print $2,$1,$3}' > white4.txt

awk -F"\t" 'BEGIN {OFS="\t"} {$1=$1"#"$2;print $1,$3}' white4.txt > white2.txt ;mv white2.txt white.txt

awk -F"\t" '{print $4}' asian.txt | unexpand -a | awk '{print $1}' > asian3.txt

cut -f1,8 asian.txt > asian2.txt

paste asian3.txt asian2.txt | awk -F"\t" 'BEGIN {OFS="\t"}{print $2,$1,$3}' > asian4.txt

awk -F"\t" 'BEGIN {OFS="\t"} {$1=$1"#"$2;print $1,$3}' asian4.txt > asian2.txt ;mv asian2.txt asian.txt

\awk -F"\t" '{print $4}' black.txt | unexpand -a | awk '{print $1}' > black3.txt

cut -f1,8 black.txt > black2.txt

paste black3.txt black2.txt | awk -F"\t" 'BEGIN {OFS="\t"}{print $2,$1,$3}' > black4.txt

awk -F"\t" 'BEGIN {OFS="\t"} {$1=$1"#"$2;print $1,$3}' black4.txt > black2.txt ;mv black2.txt black.txt

awk -F"\t" '{print $4}' pacific.txt | unexpand -a | awk '{print $1}' > pacific3.txt

cut -f1,8 pacific.txt > pacific2.txt

paste pacific3.txt pacific2.txt | awk -F"\t" 'BEGIN {OFS="\t"}{print $2,$1,$3}' > pacific4.txt

awk -F"\t" 'BEGIN {OFS="\t"} {$1=$1"#"$2;print $1,$3}' pacific4.txt > pacific2.txt ;mv pacific2.txt pacific.txt

awk -F"\t" '{print $4}' latino.txt | unexpand -a | awk '{print $1}' > latino3.txt

cut -f1,8 latino.txt > latino2.txt

paste latino3.txt latino2.txt | awk -F"\t" 'BEGIN {OFS="\t"}{print $2,$1,$3}' > latino4.txt

awk -F"\t" 'BEGIN {OFS="\t"} {$1=$1"#"$2;print $1,$3}' latino4.txt > latino2.txt ;mv latino2.txt latino.txt

awk -F"\t" '{print $4}' indian.txt | unexpand -a | awk '{print $1}' > indian3.txt

cut -f1,8 indian.txt > indian2.txt

paste indian3.txt indian2.txt | awk -F"\t" 'BEGIN {OFS="\t"}{print $2,$1,$3}' > indian4.txt

awk -F"\t" 'BEGIN {OFS="\t"} {$1=$1"#"$2;print $1,$3}' indian4.txt > indian2.txt ;mv indian2.txt indian.txt

awk -F"\t" '{print $4}' update_provider.txt | unexpand -a | awk '{print $1}' > update_provider3.txt

cut -f1,8 update_provider.txt > update_provider2.txt

paste update_provider3.txt update_provider2.txt | awk -F"\t" 'BEGIN {OFS="\t"}{print $2,$1,$3}' > update_provider4.txt

awk -F"\t" 'BEGIN {OFS="\t"} {$1=$1"#"$2;print $1,$3}' update_provider4.txt > update_provider2.txt ;mv update_provider2.txt update_provider.txt

awk -F"\t" '{print $4}' give_sample.txt | unexpand -a | awk '{print $1}' > give_sample3.txt

cut -f1,8 give_sample.txt > give_sample2.txt

paste give_sample3.txt give_sample2.txt | awk -F"\t" 'BEGIN {OFS="\t"}{print $2,$1,$3}' > give_sample4.txt

**Steps to deidentify, clean, and transfer PEER data into REDCap (cont):**

awk -F"\t" 'BEGIN {OFS="\t"} {$1=$1"#"$2;print $1,$3}' give_sample4.txt > give_sample2.txt ;mv give_sample2.txt give_sample.txt

awk -F"\t" '{print $4}' interested_research.txt | unexpand -a | awk '{print $1}' > interested_research3.txt

cut -f1,8 interested_research.txt > interested_research2.txt

paste interested_research3.txt interested_research2.txt | awk -F"\t" 'BEGIN {OFS="\t"}{print $2,$1,$3}' > interested_research4.txt

awk -F"\t" 'BEGIN {OFS="\t"} {$1=$1"#"$2;print $1,$3}' interested_research4.txt > interested_research2.txt ;mv interested_research2.txt interested_research.txt

awk -F"\t" '{print $4}' informed_me.txt | unexpand -a | awk '{print $1}' > informed_me3.txt

cut -f1,8 informed_me.txt > informed_me2.txt

paste informed_me3.txt informed_me2.txt | awk -F"\t" 'BEGIN {OFS="\t"}{print $2,$1,$3}' > informed_me4.txt

awk 'BEGIN {OFS="\t"}{$1=$1"#"$2;print $1,$3}' informed_me4.txt > informed_me2.txt ;mv informed_me2.txt informed_me.txt

awk -F"\t" '{print $4}' preferred_email.txt | unexpand -a | awk '{print $1}' > preferred_email3.txt

cut -f1,8 preferred_email.txt > preferred_email2.txt

paste preferred_email3.txt preferred_email2.txt | awk -F"\t" 'BEGIN {OFS="\t"}{print $2,$1,$3}' > preferred_email4.txt

awk 'BEGIN {OFS="\t"}{$1=$1"#"$2;print $1,$3}' preferred_email4.txt > preferred_email2.txt ;mv preferred_email2.txt preferred_email.txt

awk -F"\t" '{print $4}' preferred_postal.txt | unexpand -a | awk '{print $1}' > preferred_postal3.txt

cut -f1,8 preferred_postal.txt > preferred_postal2.txt

paste preferred_postal3.txt preferred_postal2.txt | awk -F"\t" 'BEGIN {OFS="\t"}{print $2,$1,$3}' > preferred_postal4.txt

awk 'BEGIN {OFS="\t"}{$1=$1"#"$2;print $1,$3}' preferred_postal4.txt > preferred_postal2.txt ;mv preferred_postal2.txt preferred_postal.txt

awk -F"\t" '{print $4}' preferred_text.txt | unexpand -a | awk '{print $1}' > preferred_text3.txt

cut -f1,8 preferred_text.txt > preferred_text2.txt

paste preferred_text3.txt preferred_text2.txt | awk -F"\t" 'BEGIN {OFS="\t"}{print $2,$1,$3}' > preferred_text4.txt

awk 'BEGIN {OFS="\t"}{$1=$1"#"$2;print $1,$3}' preferred_text4.txt > preferred_text2.txt ;mv preferred_text2.txt preferred_text.txt

awk -F"\t" '{print $4}' preferred_phone.txt | unexpand -a | awk '{print $1}' > preferred_phone3.txt

cut -f1,8 preferred_phone.txt > preferred_phone2.txt

paste preferred_phone3.txt preferred_phone2.txt | awk -F"\t" 'BEGIN {OFS="\t"}{print $2,$1,$3}' > preferred_phone4.txt

awk 'BEGIN {OFS="\t"}{$1=$1"#"$2;print $1,$3}' preferred_phone4.txt > preferred_phone2.txt ;mv preferred_phone2.txt preferred_phone.txt

sed -i -e 's/,//g' karyotype.txt

sed -i -e 's/Isochrome/Isochromosome/g' karyotype.txt

awk -F"\t" '{print $4}' bisexual.txt | unexpand -a | awk '{print $1}' > bisexual3.txt

cut -f1,8 bisexual.txt > bisexual2.txt

paste bisexual3.txt bisexual2.txt | awk -F"\t" 'BEGIN {OFS="\t"}{print $2,$1,$3}' > bisexual4.txt

awk -F"\t" 'BEGIN {OFS="\t"} {$1=$1"#"$2;print $1,$3}' bisexual4.txt > bisexual2.txt ;mv bisexual2.txt bisexual.txt

awk -F"\t" '{print $4}' gay.txt | unexpand -a | awk '{print $1}' > gay3.txt

cut -f1,8 gay.txt > gay2.txt

paste gay3.txt gay2.txt | awk -F"\t" 'BEGIN {OFS="\t"}{print $2,$1,$3}' > gay4.txt

awk -F"\t" 'BEGIN {OFS="\t"} {$1=$1"#"$2;print $1,$3}' gay4.txt > gay2.txt ;mv gay2.txt gay.txt

awk -F"\t" '{print $4}' straight.txt | unexpand -a | awk '{print $1}' > straight3.txt

cut -f1,8 straight.txt > straight2.txt

paste straight3.txt straight2.txt | awk -F"\t" 'BEGIN {OFS="\t"}{print $2,$1,$3}' > straight4.txt

awk -F"\t" 'BEGIN {OFS="\t"} {$1=$1"#"$2;print $1,$3}' straight4.txt > straight2.txt ;mv straight2.txt straight.txt

awk -F"\t" '{print $4}' skin_chromosome.txt | unexpand -a | awk '{print $1}' > skin_chromosome3.txt

cut -f1,8 skin_chromosome.txt > skin_chromosome2.txt

paste skin_chromosome3.txt skin_chromosome2.txt | awk -F"\t" 'BEGIN {OFS="\t"}{print $2,$1,$3}' > skin_chromosome4.txt

**Steps to deidentify, clean, and transfer PEER data into REDCap (cont):**

awk -F"\t" 'BEGIN {OFS="\t"} {$1=$1"#"$2;print $1,$3}' skin_chromosome4.txt > skin_chromosome2.txt ;mv skin_chromosome2.txt skin_chromosome.txt

awk -F"\t" '{print $4}' gentac_registry.txt | unexpand -a | awk '{print $1}' > gentac_registry3.txt

cut -f1,8 gentac_registry.txt > gentac_registry2.txt

paste gentac_registry3.txt gentac_registry2.txt | awk -F"\t" 'BEGIN {OFS="\t"}{print $2,$1,$3}' > gentac_registry4.txt

awk -F"\t" 'BEGIN {OFS="\t"} {$1=$1"#"$2;print $1,$3}' gentac_registry4.txt > gentac_registry2.txt ;mv gentac_registry2.txt gentac_registry.txt

sed -i -e 's/,//g' estrogen_treat.txt

1. **Extract unique ID – date combination field:**
   1. awk -F"\t" '{print $4}' TSRR.txt | unexpand -a | awk '{print $1}' > TSRR.IDs.txt
   2. awk -F"\t" '{print $1}' TSRR.txt > TSRR.IDs.2.txt
   3. paste TSRR.IDs.2.txt TSRR.IDs.txt > TSRR.IDs.3.tx
   4. awk '{$1=$1"#"$2;print $1,$3}' TSRR.IDs.3.txt > TSRR.IDs.2.txt
   5. sort TSRR.IDs.2.txt > TSRR.IDs.txt
   6. uniq TSRR.IDs.txt > TSRR.IDs
   7. find . -type f | awk '{print length($1) " " $1}' | sort -n | cut -d ' ' -f 2 > TSRR.IDs.2; mv TSRR.IDs.2 TSRR.IDs
2. **Create age at time of survey variable from DOB:**
   1. grep "My date of birth" TSRR.data > DOB.txt
   2. awk '{print $1,$7}' DOB.txt > DOB2.txt; mv DOB2.txt DOB.txt
   3. awk '{gsub("#","\t",$0); print;}' TSRR.IDs > TSRR.dates
   4. sort -k1,1 DOB.txt > DOB.sort;mv DOB.sort DOB.txt
   5. sort -k1,1 TSRR.dates > TSRR.sort;mv TSRR.sort TSRR.dates
   6. join TSRR.dates DOB.txt
   7. awk '{gsub("/","-",$0); print;}' age1.txt > age2.txt
   8. Open age2.txt in Excel and use YEARS() function to subtract DOB from date of survey => age3.txt
   9. awk '{$1=$1"#"$2;print $1,$4}' age3.txt > age4.txt; mv age4.txt age.txt
   10. Open age.txt in Excel and recode date format 2020-03-04 to match other variables
3. **Create TSRR data frame:**
   1. python accumulate-phenotypes.py tsrr_files/TSRR.IDs [variable_name].txt
   2. Where accumulate-phenotypes.py is:

#!/usr/bin/env python

from __future__ import print_function

import sys

def accumulate(idn, pheno_dict):

accumulation = [idn]

for pheno in sys.argv[2:]:

if idn in pheno_dict[pheno]:

accumulation.append(pheno_dict[pheno][idn])

else:

accumulation.append('NA')

return accumulation

def main():

if (len(sys.argv)) < 3:

print( 'Usage: {} <ID file> <phenotype 1> <phenotype 2> ...'.format(sys.argv[0]) )

print( 'Example: {} TSRR.IDs *.txt'.format(sys.argv[0]) )

return 1

with open(sys.argv[1], 'ro') as f:

idn_list = f.read().splitlines()

**Steps to deidentify, clean, and transfer PEER data into REDCap data frame (cont):**

pheno_dict = {}

for pheno in sys.argv[2:]:

pheno_dict[pheno] = {}

with open (pheno, 'ro') as f:

data = f.read().splitlines()

for line in data:

tmplist = line.split('\t', 1)

pheno_dict[pheno][tmplist[0]] = tmplist[1]

result = []

result.append(sys.argv[1:])

for idn in idn_list:

result.append(accumulate(idn, pheno_dict))

with open( sys.argv[1] + '.output', 'w') as o:

for line in result:

tmpstr = '\t'.join(line) + '\n'

o.write(tmpstr)

if __name__ == '__main__':

main()

1. **Edit data frame using a unix text editor:**
   1. Find and replace to remove .txt from variable names and change # to ^
   2. Change ID field to record_id
   3. Replace tabs with commas
   4. Change filename to TSRR.data.frame.csv
2. **Edit data dictionary:**
   1. Name variables exactly the same as [response].txt files in alphebetical order
   2. Add record_id as first row
   3. Change all data types to text
   4. Shorten options to < 24 characters
   5. Save as .csv file
   6. Open in Excel to check data integrity and alignment
3. **Create new REDCap project “TSSUS TSRR” as ‘support operations’ with ‘enable surveys’**
4. **Upload data dictionary into REDCap project**
5. **Upload data into REDCap project:**
   1. REDCap only allows 100 rows or subject IDs per import
   2. Split TSRR.data.frame.csv into 100-line files
      1. Tail -n +2 TSRR.data.frame.csv > headerless
      2. Head -n1 TSRR.data.frame.csv > header
      3. split -l 100 headerless part
      4. cat header partaa > partaa.csv .. partaj.csv
   3. Upload 100 line chunks into REDCap

**Visual Basic for Apps script for Outlook email redirection to TSRR survey recipients:**

Private WithEvents Items As Outlook.Items

Private Records_ As Scripting.Dictionary

Private Const XLSDataSource = "Z:\records.xlsx"

Private Sub Application_ItemSend(ByVal Item As Object, Cancel As Boolean)

End Sub

Private Sub Application_Startup()

Dim olApp As Outlook.Application

Dim objNS As Outlook.NameSpace

Set olApp = Outlook.Application

Set objNS = olApp.GetNamespace("MAPI")

' default local Inbox

Set Items = objNS.GetDefaultFolder(olFolderInbox).Items

Call GetRecords

End Sub

Private Sub Items_ItemAdd(ByVal Item As Object)

On Error GoTo ErrorHandler

If TypeName(Item) <> "MailItem" Then

Exit Sub

End If

Call GetRecords

Dim Msg As Outlook.MailItem

Set Msg = Item

Dim Subject_ As String

Subject_ = Msg.Subject

Dim Key_ As Variant

Dim Email_ As Outlook.MailItem

For Each Key_ In Records_.Keys

If InStr(1, Subject_, Key_) > 0 Then

Set Email_ = Msg.Forward

Dim Recipient_ As Outlook.Recipient

Dim To_ As String: To_ = Split(Records_(Key_), "|")(0)

Subject_ = Split(Records_(Key_), "|")(1)

Set Recipient_ = Email_.Recipients.Add(To_)

Recipient_.Type = olTo

Email_.Subject = Subject_

Email_.Recipients.ResolveAll

Email_.Send

Exit For

End If

Next

ProgramExit:

Exit Sub

ErrorHandler:

MsgBox Err.Number & " - " & Err.Description

Resume ProgramExit

End Sub

Sub GetRecords()

On Error GoTo ErrLine

Dim xlsApp As Object

Dim xlsWbk As Object

Dim xlsSht As Object

Set xlsApp = CreateObject("Excel.Application")

xlsApp.Visible = False

Set xlsWbk = xlsApp.Workbooks.Open(XLSDataSource)

Set xlsSht = xlsWbk.Sheets(1)

**Visual Basic for Apps script for Outlook email redirection to TSRR survey recipients (cont):**

Dim IntRow As Integer: IntRow = 2

Dim IDCol As Integer: IDCol = 1

Dim EmailCol As Integer: EmailCol = 2

Dim Subject_ As String

Set Records_ = CreateObject("Scripting.Dictionary")

Do While True

If xlsSht.cells(IntRow, IDCol).Text = "" Then Exit Do

Subject_ = xlsSht.cells(IntRow, EmailCol).Text & "|" & xlsSht.cells(IntRow, 6).Text

Records_.Add xlsSht.cells(IntRow, IDCol).Text, Subject_

IntRow = IntRow + 1

Loop

ErrLine:

xlsApp.Quit

Set xlsApp = Nothing

If Err <> 0 Then

MsgBox "Error:" & Err.Description

End If

End Sub
